# Vibro-Acoustic Distributed Sensing for Large-Scale Data-Driven Leak Detection on Urban Distribution Mains

**DOI:** 10.3390/s22186897

**Published:** 2022-09-13

**Authors:** Lili Bykerk, Jaime Valls Miro

**Affiliations:** Robotics Institute (UTS:RI), University of Technology Sydney, Ultimo, NSW 2007, Australia

**Keywords:** water distribution network, vibro-acoustic sensors, leak detection, structural health monitoring, feature extraction, signal processing, machine learning, binary classification, data-driven, neural network

## Abstract

Non-surfacing leaks constitute the dominant source of water losses for utilities worldwide. This paper presents advanced data-driven analysis methods for leak monitoring using commercial field-deployable semi-permanent vibro-acoustic sensors, evaluated on live data collected from extensive multi-sensor deployments across a sprawling metropolitan city. This necessarily includes a wide variety of pipeline sizes, materials and surrounding soils, as well as leak sources and rates brought about by external factors. The novel proposition for structural pipe health monitoring shows that excellent leak/no-leak classification results (>94% accuracy) can be observed using Convolutional Neural Networks (CNNs) trained with Short-Time Fourier Transforms (STFTs) of the raw audio files. Most notably, it is shown how this can be achieved irrespective of the sensor used, with four models from different manufactures being part of the investigation, and over time across extended densely populated areas.

## 1. Introduction

Potable water mains are critical components of water infrastructure. Many water utilities worldwide are managing underground pipes that have been in use for centuries. Given their age and environmental surroundings, pipes are susceptible to failures often caused by tree roots, corrosion, and/or ground movement. In addition to pipe failures, leaks can also emerge from appurtenances in the pipe network such as hydrants, valves, pipe joints, main tapping points, or service lines. Depending on the environment, water from some leaks may never surface, and will remain hidden, resulting in large water losses. When a leak becomes visible, reactive repairs are undertaken; causing disruption to customers and costly maintenance, which can be challenging for utilities to manage.

Distributed IoT sensors such as digital meters are being increasingly used by utilities to remotely monitor the performance of their network in (near) real-time. This allows the monitoring of water usage habits, and establishing the potential for leaks in the main tap and service line connection to a home. In the distribution network, IoT flow meters have been explored to identify leakage. A small experimental laboratory study contrasting various machine learning algorithms (random forest, decision trees, neural networks, and Support Vector Machine) revealed the former as the best at detecting leaks with a 75% accuracy [1]. These sensors require access to the water column to operate, a non-trivial exercise in distribution networks, thus severely limiting their leak identification and localisation capabilities. They have not been widely adopted by the industry, whose preference is for non-intrusive and portable sensing methods, such as contact acoustics-based signalling. As water discharges from a leak in the pipe network, vibrations are induced and propagated along the pipe wall. To detect hidden leaks, utilities commonly schedule Active Leak Detection (ALD) teams to periodically sweep areas of pipelines using acoustic leak detection equipment such as listening sticks and real-time correlators [2]. The success of these ALD sweeps can be hindered by the prevalence of environmental and water usage noises during the day, when the sweeps are conducted, and the experience of the user [3]. Depending on the length of the utility’s pipeline network, the time that elapses between ALD sweeps may result in hidden leaks remaining undetected for long periods of time, or missed entirely. For the continuous monitoring of the network, alternative methods of leak detection are also employed, such as Minimum Night Flow (MNF) and pressure transient analysis using existing network hardware (flow meters and pressure gauges). These methods, however, are only capable of detecting possible leakage in a given area, and will not provide any means of locating or pinpointing a leak location.

Vibro-acoustic sensing has been widely adopted by water utilities [4,5], mainly due to the relative low cost, ease of implementation, flexibility, and passive nature of the system, whereby no permanent changes to the water pipeline network are required for the technology to function. These semi-permanent devices can be used to effectively and remotely monitor the water mains for leakages—generally between 2 and 4 a.m.—when there is low network activity (the time period when MNF is calculated) and low levels of environmental noise. However, there are several challenges and uncertainties in analysing the acoustic sensor data for leak detection: (1) a leak noise can be attenuated due to fittings, joints, junctions, and service connections which are often undocumented; (2) the presence of environmental noises, and water usage in the network; (3) the signal recorded by the acoustic sensor is directly related to the pipe material and diameter, proximity to the leak noise and the quality of the sensor’s mounting point on the asset [6,7].

Semi-permanent vibro-acoustic noise loggers have in-built algorithms which raise leak alarms based on the intensity and consistency of the recorded noise [8]. Using this method, a large number of false positive leak alarms are raised by the system, and quieter leaks are missed (false negatives). By understanding the limitations of these in-built leak detection algorithms, and the uncertainties affecting the data recorded by an acoustic logger, there is a motivation and need for a more advanced analysis of the acoustic data to achieve accurate and reliable leak detection. Signal processing and data-driven machine learning methods are common techniques to increase the reliability of leak detection using vibro-acoustic noise loggers. Most leak detection approaches in the literature extract features from an audio recording, which is either directly used to interpret signals for leakage [7,9,10,11], or used to train machine learning classifiers. Models trained with simple features such as the absolute noise level recorded by loggers [12], or cross-correlation and coherence signals from neighbouring correlating noise loggers [13] have also demonstrated high accuracies in leak localisation and classification, respectively. Other methods rely on having collected baseline signals or signals before and after a leak has been repaired [14,15,16,17], to establish leak detection thresholds. Due to the persistent nature of a leak signal in an audio recording, Recurrence Plots (RPs) offer an alternative input for a binary classifier, with RPs of leak noises showing strong deterministic properties [18].

Data-driven machine learning studies have leveraged frequency-domain features of acoustic signals for training such as the Power Spectrum Density (PSD) [14,19] or Intrinsic Mode Functions (IMFs) [20]. Whilst these features may prove effective for classification in controlled laboratory tests, they are easily influenced by a temporary ambient noise which can mask a persistent leak noise in the PSD, leading to decreased classification performance [21]. This limitation is critical for sensor deployments on functioning pipeline networks, where both persistent and transient non-leak noises are prevalent, leak noises are not controlled, and the pipe network can be complex. Many of these studies are conducted in controlled laboratory environments [22,23,24,25], with few examples of data sets obtained from real pipeline networks. Data collected from in-field deployments of vibro-acoustic sensors have predominantly contained unbalanced data sets, with small amounts of leak samples [18,26,27] or data collected with minimal interference noises, where Gaussian White Noise (GWN) with different Signal-to-noise Ratios (SNRs) are added to augment the data sets [21]. Unbalanced data sets remain a limitation in evaluating the success of any leak detection classifier, particularly for real-world sensor deployments where pipe materials, diameters, soil properties, service lines, and offtakes, amongst other geospatial features, can vary significantly and heavily influence the signals recorded by the vibro-acoustic sensors.

Time–frequency features generated using discrete Short-time Fourier Transforms (STFTs), such as spectrograms, reveal the temporal nature of a signal that is not captured by analysing frequency-domain features alone. STFTs can provide rich features for machine learning; however, STFTs as standalone input features are rarely used for acoustic signal analysis, due to a limitation in the time–frequency resolution [28]. In an effort to balance the relationship between the time and frequency resolutions, a Time–Frequency Convolutional Neural Network (TFCNN), with three different spectrogram resolutions as inputs is proposed to study the efficacy of classification under varying SNR conditions in real pipeline networks [21]. The TFCNN model is compared against a range of other common classifiers, including a CNN trained with Fast Fourier Transform (FFT) data (Frequency Convolutional Neural Network (FCNN)). It is reported that the spectrogram contains sufficient defining characteristics of a leak signal (as opposed to time, or frequency-based features alone), and is therefore more favourable and reliable as an input to a leak detection system. Mel-frequency spectrograms, which closely align with the human perception of sound, are also commonly used as features in machine learning applications, including leak classification problems [29,30].

This paper evaluates state-of-the-art data-driven methods for leak classification using data collected from semi-permanent vibro-acoustic logger deployments in small reticulation mains across metropolitan Sydney over the course of up to 24 months. Data from a range of commercially available types of vibro-acoustic sensors deployed in different metropolitan areas of a utility-managed water network are used to evaluate the efficacy of existing data-driven methods (FCNN and TFCNN models [21]) for reliable leak detection in urban distribution mains.

The paper is organised as follows. Section 2 details the vibro-acoustic sensors and data loggers, data collection, signal processing, data curation, feature extraction and binary classification methods. Section 3 presents the results and discussion. Finally, the conclusions and future work are presented in Section 4.

## 2. Materials and Methods

### 2.1. Vibro-Acoustic Sensors and Data Loggers

Vibro-acoustic logging hardware consists of a vibro-acoustic sensor, data logger, and other peripherals such as GSM transmitters and antennas to send the collected data to the cloud. Vibro-acoustic sensors function on the premise that when water leaks through a pipe it creates vibrations due to the pressure differential between the inside and the outside of a pipe. The waves can travel thorough both pipe material and water, allowing the sensors to measure the vibration inflicted on the material, or directly in the water column. Standard manufacturer specifications indicate that vibro-acoustic sensors are effective in recording leakage noises on reticulation mains typically smaller than 375 mm in diameter, and can correlate over distances of up to 150 m between adjacent loggers.

**Figure 1:**
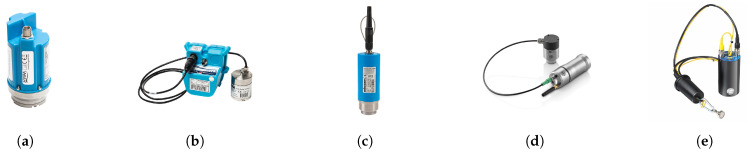
Range of vibro-acoustic loggers installed across metropolitan Sydney: (**a**) HWM PermaNET SU, (**b**) HWM PermaNET+, (**c**) SebaKMT Sebalog N-3, (**d**) Von Roll ORTOMAT-MTC, (**e**) Primayer Enigma3m.

**Figure 2:**
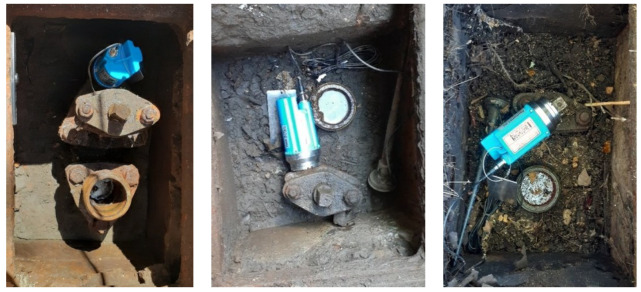
HWM PermaNET SU loggers deployed on hydrant control valves in different locations.

In December 2019, a range of vibro-acoustic sensors deployments commenced across six Central Business District (CBD) areas in metropolitan Sydney (summarised in Table 1). In these CBD areas, five different types of commercially available semi-permanent vibro-acoustic loggers (see Figure 1) were deployed. These could not be collocated in the same spots to compare performance given the chamber’s physical limitations, and the extent of exposed asset to mount them on (see some examples Figure 2), and were thus distributed to cover separate areas and zones (when within the same area). It should also be noted that, given the attachment coupling of the sensor to the appurtenance, they can not physically measure the exact same point regardless, so arranging them over an extended geographical coverage of the city is more representative of a realistic deployment in a practical sense for comparison, and more effective to search for as many leaks as possible over a given time period for a more robust validation of the proposed scheme.

Each of the five different vibro-acoustic sensors and data loggers are functionally equivalent, whereby vibrations in the pipeline network are detected by the sensors and recorded with the data logging hardware. The key differences between the loggers are the quality of the hardware used, the level of processing of the data, both on the logger itself and the cloud-based portals, and the user programmable settings (e.g., audio recording duration and time).

The sensors have mostly been installed on appurtenances (valves and hydrants) attached to Cast Iron Cement Lined (CICL) or Steel Cement Lined (SCL) pipelines, ranging in diameter from 100 mm to 450 mm and up to more than 100 years old. Depending on the available space in a hydrant or valve chamber and the condition of the assets, the sensors are often mounted with differing orientations and mounting points, as shown in Figure 2.

### 2.2. Data Collection

Noises in the pipe network are measured every day at a time of low water usage and theoretically low environmental noise (between 2–4 a.m.). With the exception of the Sebalog N-3 vibro-acoustic sensors, all of the deployed sensors were programmed to record a 10-s duration audio file daily. The Sebalog N-3 units have limited configuration settings, thus, despite recording a 2.5 s duration audio clip every day, the audio file is only sent to the cloud if the logger itself determines that a leak is present (through a noise level threshold algorithm). In addition to audio recordings, other noise-level data are also available for analysis from most of the loggers; however, these were not used in this study. All loggers are equipped with integrated modems and transmit data to the cloud, with the raw acoustic data (audio files) available through the sensor manufacturers FTP servers, or accessible through API calls.

**Figure 3:**
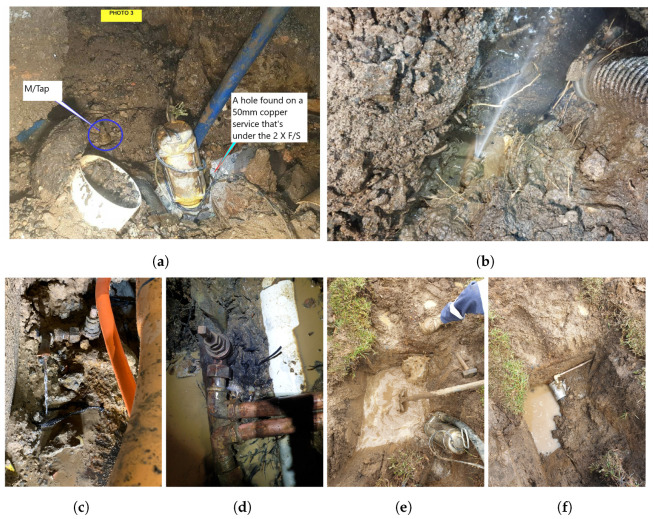
Examples of detected hidden leaks from a range of vibro-acoustic sensors and deployment areas (pictures supplied by utility field crews, taken during repairs): (**a**) Copper service leak, (**b**) Main tap leak, (**c**) Main tap leak (clamped service line), (**d**) Leak on main tap coupling piece, (**e**) leaking main tap (excavation site), and (**f**) leaking main tap repaired with full circumference pipe clamp.

The collected data consist of ’leak’ and ’no-leak’ audio recordings originating from a range of leak sources across the six deployment areas. Approximately 70% of the detected leaks were hidden, many of which were in built-up areas and estimated to have been present for up to 10 years. The detected leaks were found to have emerged from a range of sources, including hydrants ∼30%, valves ∼20%, main taps ∼22%, private ∼11%, service lines ∼12%, mains (leaks/breaks) ∼2.5%, and meter taps ∼2.5%. Some examples of hidden leaks detected by the vibro-acoustic sensors are shown in Figure 3.

The four logger data sets (HWM, Von Roll, SebaKMT, Primayer) mostly include loggers that recorded leak noises from the first day they were deployed. These existing leaks were monitored for several days to confirm the likelihood of the presence of a leak, prior to raising these locations for in-field investigation by the water utility. The leaks were confirmed on-site by skilled network technicians through use of listening sticks and pinpointed using real-time correlators. Significant delays were experienced with some repair jobs, due to the complex locations of some leaks. Consequently, many of the recorded leak signals contain the same underlying persistent leak noise, occasionally overlaid with transient environmental noises. As existing leaks were gradually repaired and baseline noise levels could be achieved, the emergence and evolution of new leaks were able to be identified and the data sets grew further in size over the course of the deployments. Since only a small subset of all of the deployed loggers detected leaks, only these loggers were included in the data sets (both before and after leak repairs), to ensure a relative balance of the data sets. To improve on the robustness of the classification in the presence of other environmental noises, those loggers which only recorded ’no-leak’ signals for the duration of their deployment could also be used.

### 2.3. Data Analysis-Signal Processing

**Figure 4:**
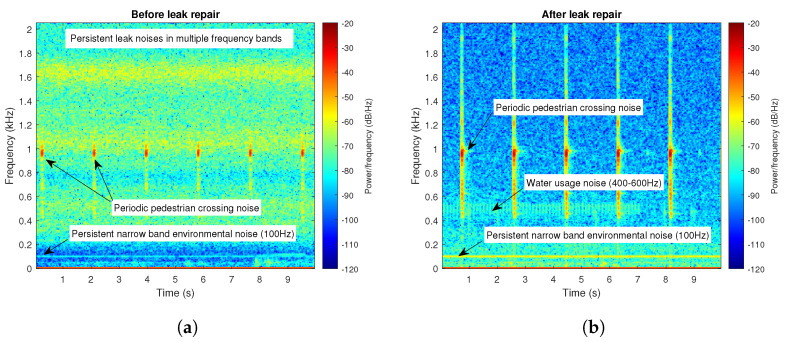
Spectrograms for a sensor detecting a variety of noises: (**a**) before and (**b**) after a leak was repaired ∼21 m away from the sensor. The sensor was situated <1 m from a pedestrian crossing.

Across the six deployment areas, a wide variety of leak noises were recorded. Some sensors were located very close to the leak source, and others at a distance, with variations in pipe diameters and materials, and several offtakes between. Using STFT signal processing techniques, acoustic signals can be best visualised by generating spectrograms, which reveal temporal changes to the frequency and power of a signal. If the audio recording contains persistent noise, without the presence of any intermittent external noises, PSD line plots can also provide a simple means of signal comparison. As leaks are continuous noise sources, their higher-power frequencies are persistent in the spectrum, for the duration of an audio recording. On the other hand, non-leak noises—such as those from environmental sources, or water usage—are mostly transient in nature, with intermittent frequency components. Some environmental noises, however, can be persistent, such as mechanical or electrical equipment which commonly emit high-power, low-frequency noises usually with narrow frequency bands. Due to these characteristic features, persistent and intermittent ’no-leak’ signals are easily distinguishable from ’leak’ signals in a spectrogram (see Figure 4 for an example). Due to the close coupling of the sensors to the water main, leaks generally have a distinguishing pattern in the audio spectrum, even in the presence of other intermittent noises.

**Figure 5:**
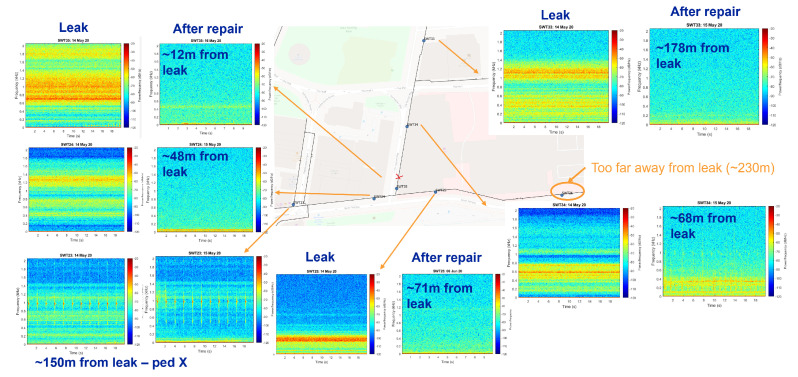
An illustration of a single leak source originating from a broken back pipe, and the noise spectrograms picked up by 6 HWM PermaNET+ loggers in the vicinity. The 7th sensor on the right is located too far away to pick it up.

By clustering the loggers in the pipeline networks to ensure neighbouring loggers are able to correlate, often more than one logger was able to record noise from a single leak source; one such example is shown in Figure 5, where six vibro-acoustic sensors were able to detect the leak noise caused by a broken back on the pipe (main break). The shift in the dominant leak frequency can be observed with increased distance between the leak and the sensor. Other contributing factors to the frequency shift could also include pipe material change and junctions and offtakes between the leak and the sensor. In general, the further away the sensor is from the leak location, the more the higher-frequency components of the spectrum are attenuated, and the lower frequency noises are more prevalent. With increased distance between the logger and the noise source, the intensity (power) of the noise also decays.

**Figure 6:**
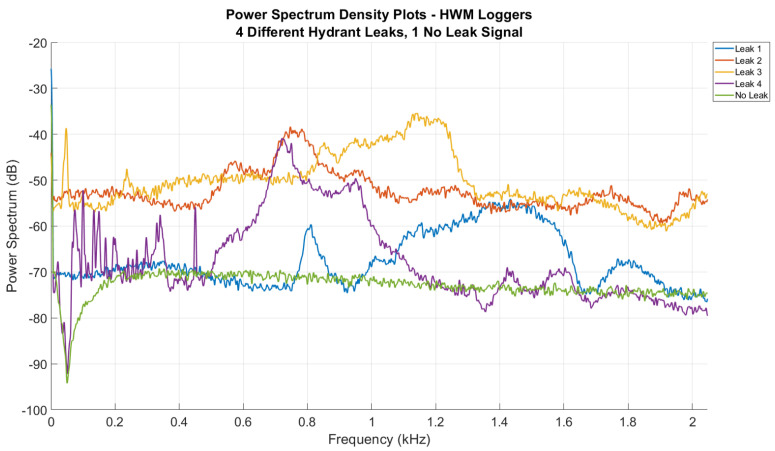
PSD plot: four different hydrant leak signals (loggers on leaking hydrants).

A leak located close to the hydrant where the logger is installed will typically have elevated noise across the spectrum, often with higher power in high frequency band/s. Figure 6 shows PSD line plots from HWM vibro-acoustic sensors detecting leaks at the hydrant they were installed on. All leaks were on screw-down-type hydrants, and suspected to be of varying leak rates. The vibro-acoustic sensors were installed in different orientations and with different contact points on the hydrants, similar to those mounting configurations shown in Figure 2. There is a significant difference in the PSDs of each hydrant leak. The difference in signals could be attributed to many factors including the quality of the attachment point of the sensor on the asset or the magnitude of the leak. Comparing these signals to a ’quiet’, baseline signal with no leak present, it is noted that all four leak signals show elevated power across almost all recorded frequencies, and clear peaks in the spectrum at certain frequencies. This indicates that despite leaking hydrant signals being inconsistent across multiple loggers/hydrants, there is still a significant deviation from a baseline ’no-leak’ noise that is sufficient to detect a leak.

### 2.4. Data Analysis-Data Curation

In order to curate the collected data to train machine learning classifiers, the raw acoustic data were analysed in the time, frequency, and time–frequency domains using the signal processing and visualisation techniques (PSD, STFT, FFT) described in Section 2.3. Analysis of the vibro-acoustic data, in conjunction with feedback from the utility field crews, allowed for a database to be compiled with key information pertaining to the leaks. The collated and curated data consist of the audio file name, date of audio recording and binary class label (’leak’ or ’no-leak’). Other collected information not used for the binary classification includes the leak source, and the distance, pipe material/s, and diameter/s between the leak and logger.

**Figure 7:**
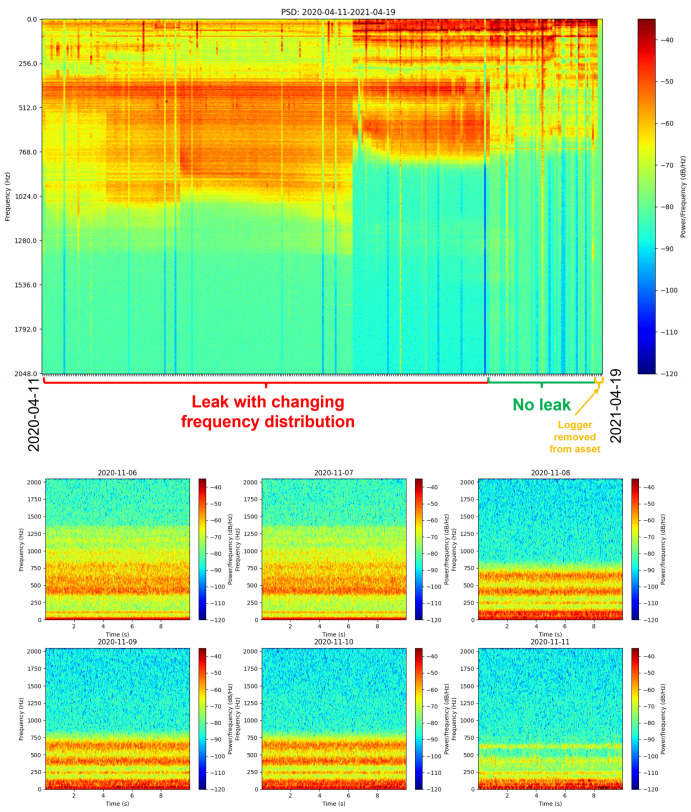
Leaking main tap-changing frequency distribution is visible in the PSD (**top**) and spectrograms (**bottom**) from consecutive days. The logger was situated approximately 59 m away from the leak location, with noise being propagated along a straight section of 150 mm diameter CICL pipe.

Most of the detected leaks were present prior to the loggers being installed; however, there were some instances where new leaks emerged during the logger deployment time. For the leaks that were already present, the collected acoustic signals were generally stable and unchanged in their frequency. In some instances, noticeable frequency/power shifts in the spectrum were observed (see Figure 7)—possibly from a leak worsening, or the sensor being slightly shifted/dislodged on the asset due to environmental factors or human intervention. These cases were carefully analysed to ensure that the data was representative of a true ’leak’ or ’no-leak’ signal, and the logger had not been dislodged from the asset.

The curated data from individual loggers were compiled into complete data sets for each logger *manufacturer* (for a total of four discrete data sets). Due to the slightly differing frequency ranges and audio recording duration (as listed in Table 1), individual classifiers were trained for each sensor manufacturer and were evaluated individually. With nearly 300 loggers deployed across the six deployment areas over the course of two years, the complete data sets from each logger manufacturer are vast. To ensure a relative balance of data for each data set, only data from loggers which recorded both ’leak’ and ’no-leak’ signals throughout their deployment are included in the data sets.

### 2.5. Feature Extraction and Binary Classification

To evaluate the performance of a binary classifier for each of the data sets, an extensive literature review on the topic of data-driven leak detection methods with acoustic data was first conducted. A critical criteria in determining the suitability of a classifier was the reported performance with data collected from real pipeline networks. With limited studies and evaluations utilising data from deployments of loggers outside of controlled laboratory environments, it was found that CNN-based classifiers leveraging features obtained from FFTs and STFTs (spectrograms) had the best reported performance, compared with other common binary classification models.

**Figure 8:**
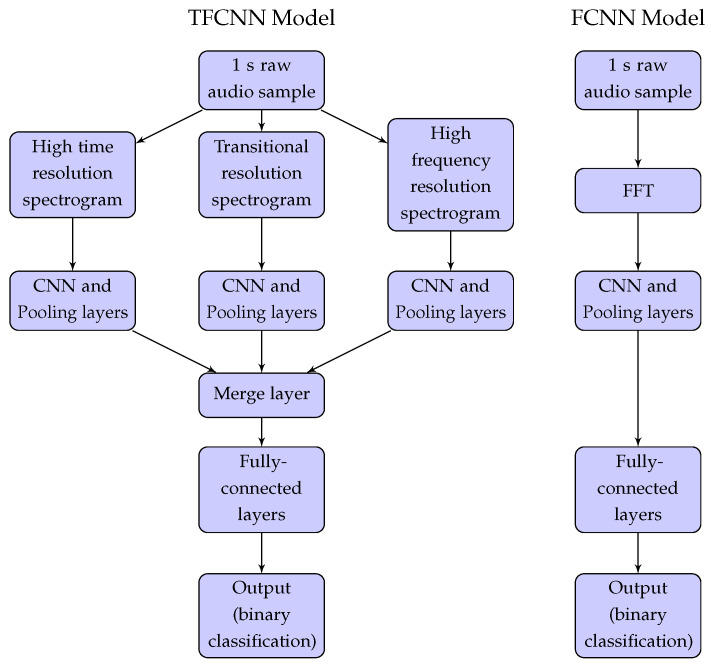
TFCNN and FCNN model structures.

Both the FCNN and TFCNN models from [21] are trained and evaluated in this paper, using the four discrete data sets collected from the six deployment areas. The data sets were first prepared by augmenting [31] (splitting) each audio file into several 1 s audio chunks. For the SebaKMT loggers, only the first two seconds of the 2.5 s duration audio recordings were used. All other loggers (with 10 s duration) audio recordings were split into 10 individual audio chunks. Due to the vast array of samples, including various ’no-leak’ noise sources, it was not deemed necessary to further augment the data sets by adding GWN with different SNRs into the raw signals. To extract the frequency bands of interest where leaks are most common, the 1 s duration audio samples are also bandpass filtered (100–2000 Hz). With the data sets collected and curated, finally, a random 80% of each complete data set (for each logger type) was used for training and 20% for testing. The models (whose structures are shown in Figure 8) were implemented in Python 3.9 using Keras [32] and TensorFlow [33] version 2.6.0.

The input to the FCNN model is purely frequency-domain based—a FFT of the 1 s audio signal. The inputs to the TFCNN model are three spectrograms generated from the same 1 s audio signal. Each spectrogram is generated with a different time–frequency resolution (high time, transitional, high frequency) and is intended to improve the leak detection performance, since ’no-leak’ and ’leak’ noises have different time–frequency components. A high-time-resolution spectrogram reflects the change of the signal in the time-domain, where a leak signal is most stable. In these spectrograms, the presence of any transient noises are most obvious. The high-frequency-resolution spectrogram reflects the spectral structure and energy distribution of the signal in the frequency domain. Whilst transient noises can still be observed in these spectrograms, the leak frequency or frequencies are best represented. Finally, the transitional time–frequency resolution is intended to balance the relationship between the time and frequency resolutions. Due to different sampling rates of the four sets of loggers, the dimensions of the three spectrograms which are the inputs for the TFCNN model differ slightly, as listed in Table 2.

## 3. Results and Discussion

Table 3 and Table 4 summarise the results of the FCNN and TFCNN classification models for the four logger data sets. The metrics used to evaluate the model performance were accuracy, sensitivity, and specificity. The following abbreviations are used to simplify the presentation of the equations: True Positive (*TP*); True Negative (*TN*); False Positive (*FP*); False Negative (*FN*). Accuracy is the measure of the classifier’s overall correct classification performance: TP+TN/(TP+TN+FP+FN). Sensitivity is the classifier’s ability to label a ’leak’ signal as ’leak’ (recall of the positive class): TP/(TP+FN). Specificity is the classifier’s ability to label a ’no-leak’ signal as ’no-leak’ (recall of the negative class): TN/(TN+FP).

Despite the excellent performance of the FCNN model, as was reported in [21], it was found that the TFCNN model consistently outperformed the FCNN model across each of the performance metrics studied (with the exception of the specificity of the HWM loggers). This indicates that the spectrogram-based inputs are more effective than purely frequency-domain-based inputs in representing the characteristics of both ’leak’ and ’no-leak’ signals for binary classification.

**Figure 9:**
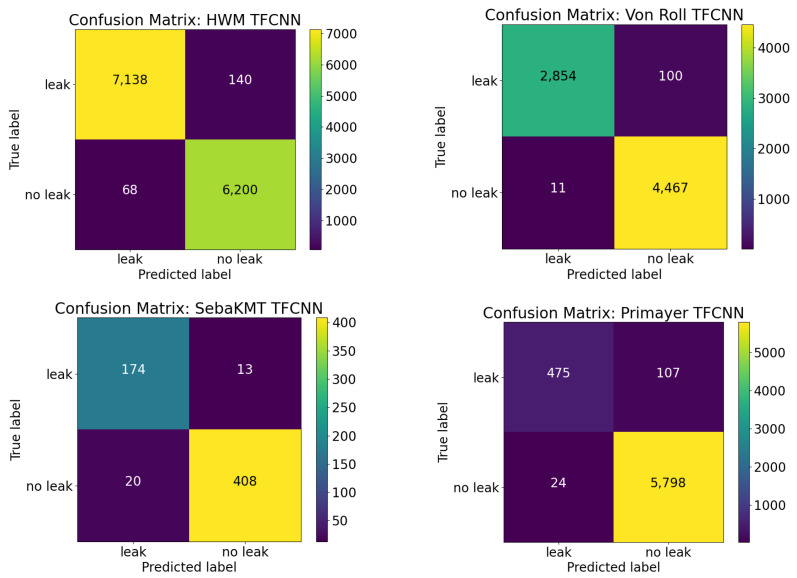
Confusion matrices for TFCNN models. HWM (**top left**), Von Roll (**top right**), Seba KMT (**bottom left**) and Primayer (**bottom right**).

**Figure 10:**
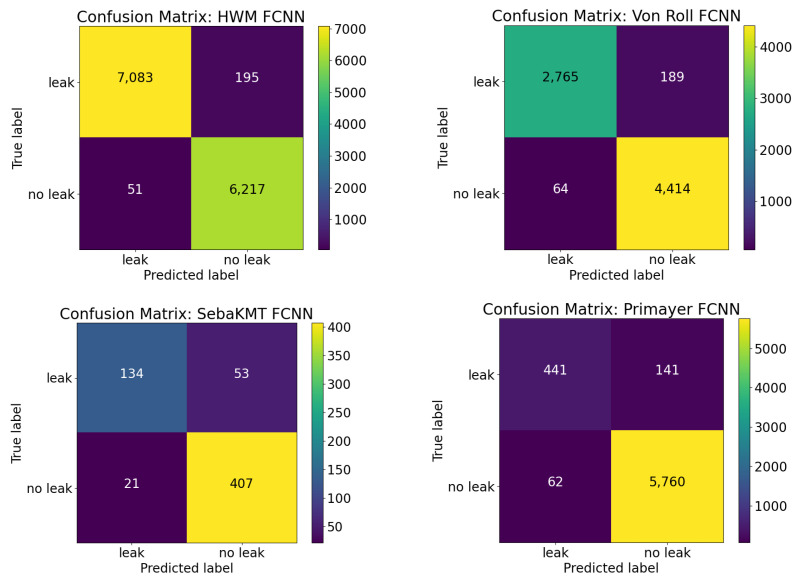
Confusion matrices for FCNN models. HWM (**top left**), Von Roll (**top right**), Seba KMT (**bottom left**) and Primayer (**bottom right**).

Figure 9 and Figure 10 show the confusion matrices for each of the four different TFCNN and FCNN trained models, respectively. For a practical leak detection system that water utilities can rely on, high accuracy but also high specificity (true negative) and sensitivity (true positive) rates are key performance metrics. A reliable leak detection system will minimise the false positive leak alarms, to ensure that any follow-up field investigations are for real leak events, maximising the efficiency for utilities.

Despite the limited data available from SebaKMT Sebalog N-3 loggers and a data imbalance with ’leak’ and ’no-leak’ signals across 3/4 of the data sets, the results indicate that the type of sensor used (different vibro-acoustic sensor with different sampling rate, sensitivity, etc.) does not affect the performance of the classifier. Furthermore, the results demonstrate that a leak detection system using either the FCNN or TFCNN model can be effectively trained with data from a single location both before and after a leak repair.

The excellent classification results show that—irrespective of the type of vibro-acoustic sensor used—the classifiers have been able to learn sufficiently with data from a range of deployment areas, where leak sources, pipe sizes and materials as well as soil conditions have varied widely. The results indicate that this is particularly relevant for identifying leaks in built-up CBD areas, where a variety of ’no-leak’ persistent and transient environmental noises are prevalent, even in the early hours of the morning. Considering all of the factors that affect the recorded vibro-acoustic signals, the results presented show great promise for water utilities looking to integrate the use of semi-permanent vibro-acoustic sensors into their business-as-usual practice for structural pipe health monitoring. Through the use of vibro-acoustic sensors and early detection of hidden leaks, proactive maintenance can be scheduled and conducted, with minimal impact to the customer.

The classification performance may be improved by including a large number of ’no-leak’ signals from elsewhere in the pipeline network during a deployment i.e., by including those other loggers that did not record both ’leak’ and ’no-leak’ signals in the data set. This will help further train the classifier to better discriminate between ’leak’ and ’no-leak’ noises, further increasing the reliability and robustness of the classification.

## 4. Conclusions

This paper studied and analysed the performance of a range of different semi-permanent vibro-acoustic sensors deployed in six CBD areas across wider Sydney for extended periods of time. Following careful collation, analysis and curation of the collected acoustic data, two state-of-the-art CNN-based classification models (FCNN and TFCNN) were trained and tested for each of the four logger types.

The results presented point towards the potency of FFT and STFT signal processing for CNN-based classification of vibro-acoustic measurements. Moreover, they represent the first known documented comparison of a variety of different semi-permanent sensing hardware, with a special underscore on the study having been undertaken on live deployments. The results demonstrate that these state-of-the-art methods are not only applicable to one particular make and model of semi-permanent acoustic sensor, as was previously documented in the single relevant case study found in the literature. Classification accuracies in the range of [94.63–98.51%] were achieved with the best performer, the TFCNN model, for all the sensors studied.

Future work to enhance the results of this study would involve obtaining further validated data collected from a wider variety of deployment locations and CBD areas. As indicated in Section 3, the robustness and reliability of these classifiers may also be improved by adding further existing ’no-leak’ audio recordings into the data sets. Finally, despite their sensing hardware similarities, a comparison of the classification performance of semi-permanent and Lift and Shift (L&S) vibro-acoustic sensors (intended for short-term deployments, rather than continuous monitoring) would also provide further insights into the potential success and value of implementing smart leak detection methods for utilities.

## Figures and Tables

**Table 1:** Vibro-Acoustic Logger Deployment Details.

Manufacturer	Model	Deployment Area #	Frequency Range (Hz)	Sampling Rate (Hz)	Audio Recording Duration (s)	# Leaks Detected
HWM	PermaNET+	1,2,3	0–2048	4096	10	19
HWM	PermaNET SU	1	0–2048	4096	10	18
Von Roll	ORTOMAT-MTC	2,4	0–2340	4681	10	16
SebaKMT	Sebalog N-3	1,5	0–3277	6554	2.5	23
Primayer	Enigma3m	4,6	0–2500	5000	10	11

**Table 2:** TFCNN model spectrogram matrix sizes for different resolutions.

Logger Manufacturer	Audio Sampling Rate (Hz)	Spectrogram Resolution		
		High Time	Transitional	High Frequency
HWM	4096	[94, 60]	[186, 28]	[372, 12]
Von Roll	4681	[99, 70]	[197, 33]	[394, 15]
SebaKMT	6554	[72, 99]	[142, 48]	[283, 22]
Primayer	5000	[94, 75]	[184, 36]	[369, 16]

**Table 3:** FCNN Results.

Logger Manufacturer	Total # Files	# Leak Files	# No Leak Files	Accuracy (%)	Specificity (%)	Sensitivity (%)
HWM	67,730	36,310	31,420	98.18	99.19	97.32
Von Roll	37,160	14,850	22,310	96.60	98.57	93.60
Seba KMT	3072	1026	2046	87.97	95.09	71.66
Primayer	32,020	2950	29,070	96.83	98.94	75.77

**Table 4:** TFCNN Results.

Logger Manufacturer	Total # Files	# Leak Files	# No Leak Files	Accuracy (%)	Specificity (%)	Sensitivity (%)
HWM	67,730	36,310	31,420	98.46	98.92	98.07
Von Roll	37,160	14,850	22,310	98.51	99.75	96.61
Seba KMT	3072	1026	2046	94.63	95.33	93.05
Primayer	32,020	2950	29,070	97.95	99.59	81.62

## Data Availability

The data presented in this study cannot be made publicly available due to confidentiality; readers should contact the corresponding author for details.

## Abbreviations

The following abbreviations are used in this manuscript:

ALD	Active Leak Detection
API	Application Programming Interface
CBD	Central Business District
CICL	Cast Iron Cement Lined
CNN	Convolutional Neural Network
FCNN	Frequency Convolutional Neural Network
FFT	Fast Fourier Transform
FTP	File Transfer Protocol
GSM	Global System for Mobile communication
GWN	Gaussian White Noise
IMF	Intrinsic Mode Function
L&S	Lift and Shift (vibro-acoustic sensors)
MNF	Minimum Night Flow
PSD	Power Spectrum Density
RP	Recurrence Plot
SCL	Steel Cement Lined
SNR	Signal-to-noise Ratio
STFT	Short-time Fourier Transform
TFCNN	Time–Frequency Convolutional Neural Network

## References

[1] Alves Coelho J., Glória A., Sebastião P. (2020). Precise Water Leak Detection Using Machine Learning and Real-Time Sensor Data. IoT.

[2] Hamilton S., Charalambous B. (2013). Leak Detection: Technology and Implementation.

[3] Hunaidi O., Wang A., Bracken M., Gambino T., Fricke C. Acoustic methods for locating leaks in municipal water pipe networks. Proceedings of the International Conference on Water Demand Management.

[4] Age of Data-driven Leak Management Dawns. Water Online. www.wateronline.com/doc/age-of-data-driven-leak-management-dawns-0001.

[5] Noise Loggers–The Technology Which Is Transforming Leak Detection for the United Kingdom’s Pipe Network. piperepair.co.uk/2020/06/30/noise-loggers-the-technology-which-is-transforming-leak-detection-for-the-united-kingdoms-pipe-network/.

[6] Fuchs H.V., Riehle R. (1991). Ten Years of Experience with Leak Detection by Acoustic Signal Analysis. Appl. Acoust..

[7] Gong J., Lambert M.F., Stephens M.L., Cazzolato B.S., Zhang C. (2020). Detection of Emerging through-Wall Cracks for Pipe Break Early Warning in Water Distribution Systems Using Permanent Acoustic Monitoring and Acoustic Wave Analysis. Water Resour. Manag..

[8] Pilcher R., Hamilton S., Chapman H., Field D., Ristovski B., Stapely S. (2007). Leak Location and Repair: Guidance Notes.

[9] Stephens M., Gong J., Zhang C., Marchi A., Dix L., Lambert M.F. (2020). Leak-Before-Break Main Failure Prevention for Water Distribution Pipes Using Acoustic Smart Water Technologies: Case Study in Adelaide. J. Water Resour. Plann. Manag..

[10] Marmarokopos K., Doukakis D., Frantziskonis G., Avlonitis M. (2018). Leak Detection in Plastic Water Supply Pipes with a High Signal-to-Noise Ratio Accelerometer. Meas. Control.

[11] Martini A., Troncossi M., Rivola A. (2015). Automatic Leak Detection in Buried Plastic Pipes of Water Supply Networks by Means of Vibration Measurements. Shock Vib..

[12] El-Abbasy M.S., Mosleh F., Senouci A., Zayed T., Al-Derham H. (2016). Locating Leaks in Water Mains Using Noise Loggers. J. Infrastruct. Syst..

[13] Burtea V., Murray P. How Well Can Machine Learning Support Pipeline Leak Monitoring? In Proceedings of the Pipelines 2021, Virtual Conference, 3–6 August 2021.

[14] El-Zahab S., Asaad A., Abdelkader E.M., Zayed T. (2017). Collective Thinking Approach for Improving Leak Detection Systems. Smart Water.

[15] Cody R.A., Tolson B.A., Orchard J. (2020). Detecting Leaks in Water Distribution Pipes Using a Deep Autoencoder and Hydroacoustic Spectrograms. J. Comput. Civ. Eng..

[16] Martini A., Troncossi M., Rivola A. (2017). Leak Detection in Water-Filled Small-Diameter Polyethylene Pipes by Means of Acoustic Emission Measurements. Appl. Sci..

[17] Tariq S., Bakhtawar B., Zayed T. (2022). Data-driven application of MEMS-based accelerometers for leak detection in water distribution networks. Sci. Total Environ..

[18] Nam Y.W., Arai Y., Kunizane T., Koizumi A. (2021). Water leak detection based on convolutional neural network using actual leak sounds and the hold-out method. Water Supply.

[19] Teruhi S., Yamaguchi Y., Akahani J. (2017). Water Leakage Detection System for Underground Pipes by Using Wireless Sensors and Machine Learning. J. Disaster Res..

[20] Butterfield J.D., Meyers G., Meruane V., Collins R.P., Beck S.B.M. (2018). Experimental investigation into techniques to predict leak shapes in water distribution systems using vibration measurements. J. Hydroinf..

[21] Guo G., Yu X., Liu S., Ma Z. (2021). Leakage Detection in Water Distribution Systems Based on Time–Frequency Convolutional Neural Network. J. Water Resour. Plann. Manag..

[22] El-Zahab S., Abdelkader E.M., Zayed T. (2018). An accelerometer-based leak detection system. Mech. Syst. Sig. Process..

[23] Ismail M.I.M., Dziyauddin R.A., Salleh N.A.A., Muhammad-Sukki F., Bani N.A., Izhar M.A.M., Latiff L.A. (2019). A Review of Vibration Detection Methods Using Accelerometer Sensors for Water Pipeline Leakage. IEEE Access.

[24] Martini A., Rivola A., Troncossi M. (2018). Autocorrelation analysis of vibroacoustic signals measured in a test field for water leak detection. Appl. Sci..

[25] Chi Z., Li Y., Wang W., Xu C., Yuan R. (2021). Detection of water pipeline leakage based on random forest. J. Phys. Conf. Ser..

[26] Ravichandran T., Gavahi K., Ponnambalam K., Burtea V., Jamshid Mousavi S. (2021). Ensemble-based machine learning approach for improved leak detection in water mains. J. Hydroinf..

[27] Tijani I.A., Abdelmageed S., Fares A., Fan K.H., Hu Z.Y., Zayed T. (2022). Improving the leak detection efficiency in water distribution networks using noise loggers. Sci. Total Environ..

[28] Ahadi M., Bakhtiar M.S. (2010). Leak detection in water-filled plastic pipes through the application of tuned wavelet transforms to Acoustic Emission signals. Appl. Acoust..

[29] Chuang W., Tsai Y., Wang L. Leak Detection in Water Distribution Pipes Based on CNN with Mel Frequency Cepstral Coefficients. Proceedings of the 3rd International Conference on Innovation in Artificial Intelligence.

[30] Müller R., Illium S., Ritz F., Schröder T., Platschek C., Ochs J., Linnhoff-Popien C. Acoustic Leak Detection in Water Networks. Proceedings of the 13th International Conference on Agents and Artificial Intelligence (Volume 2).

[31] Kang J., Park Y., Lee J., Wang S., Eom D. (2018). Novel Leakage Detection by Ensemble CNN-SVM and Graph-Based Localization in Water Distribution Systems. IEEE Trans. Ind. Electron..

[32] Chollet F. Keras. github.com/fchollet/keras.

[33] Abadi M., Barham P., Chen J., Chen Z., Davis A., Dean J., Devin M., Ghemawat S., Irving G., Isard M. Tensorflow: A System for Large-scale Machine Learning. Proceedings of the 12th USENIX Symposium on Operating Systems Design and Implementation (OSDI 16).

